# Synaptic Organization of VGLUT3 Expressing Low-Threshold Mechanosensitive C Fiber Terminals in the Rodent Spinal Cord

**DOI:** 10.1523/ENEURO.0007-19.2019

**Published:** 2019-02-14

**Authors:** Max Larsson, Jonas Broman

**Affiliations:** Department of Clinical and Experimental Medicine, Division of Neurobiology, Linköping University, Linköping S-581 85, Sweden

**Keywords:** C-tactile, pain, spinal cord, tyrosine hydroxylase, ultrastructure

## Abstract

Low-threshold mechanosensitive C fibers (C-LTMRs) that express the vesicular glutamate transporter VGLUT3 are thought to signal affective touch, and may also play a role in mechanical allodynia. However, the nature of the central termination of C-LTMRs in the dorsal horn remains largely unexplored. Here, we used light and electron microscopy in combination with VGLUT3 immunolabeling as a marker of C-LTMR terminations to investigate this issue. VGLUT3^+^ C-LTMRs formed central terminals of Type II glomeruli in the inner part of lamina II of the dorsal horn, often establishing multiple asymmetric synapses with postsynaptic dendrites but also participating in synaptic configurations with presynaptic axons and dendrites. Unexpectedly, essentially all VGLUT3^+^ C-LTMR terminals showed substantial VGLUT1 expression in the rat, whereas such terminals in mice lacked VGLUT1. Most VGLUT3^+^ C-LTMR terminals exhibited weak-to-moderate VGLUT2 expression. Further, C-LTMR terminals formed numerous synapses with excitatory protein kinase Cγ (PKCγ) interneurons and inhibitory parvalbumin neurons, whereas synapses with calretinin neurons were scarce. C-LTMR terminals rarely if ever established synapses with neurokinin 1 receptor (NK1R)-possessing dendrites traversing lamina II. Thus, VGLUT3^+^ C-LTMR terminals appear to largely correspond to neurofilament-lacking central terminals of Type II glomeruli in inner lamina II and can thus be identified at the ultrastructural level by morphological criteria. The participation of C-LTMR terminals in Type II glomeruli involving diverse populations of interneuron indicates highly complex modes of integration of C-LTMR mediated signaling in the dorsal horn. Furthermore, differences in VGLUT1 expression indicate distinct species differences in synaptic physiology of C-LTMR terminals.

## Visual Abstract

**Figure:**
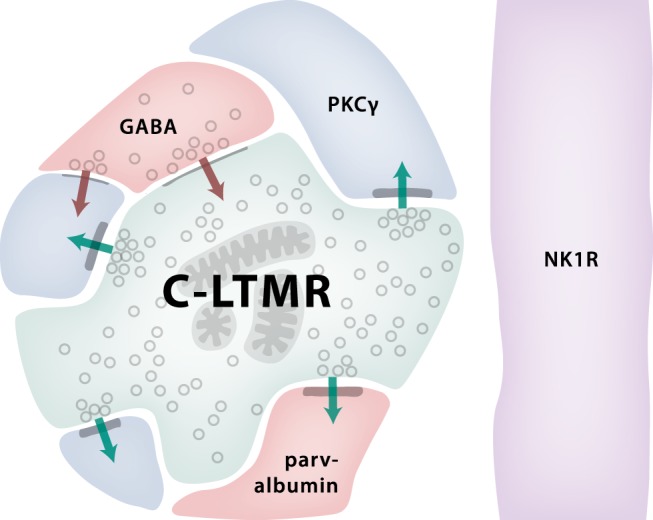

## Significance Statement

Here, we show that low-threshold mechanosensitive C fibers (C-LTMRs) form central terminals of a certain class of synaptic glomeruli, where they are subject to presynaptic inhibition and establish synapses onto distinct populations of excitatory and inhibitory interneurons in the dorsal horn. These results prompt a revised interpretation of dorsal horn ultrastructure, and provide a basis for ultrastructural identification of C-LTMRs in future studies of the role of these fibers in somatosensation. Furthermore, our observations indicate that C-LTMR terminations are subject to complex regulation and are well positioned to participate in integration of afferent signals that lead to percepts of affective touch and pain.

## Introduction

Unmyelinated C fibers that are activated by innocuous mechanical stimuli of the hairy skin were discovered in the cat nearly 80 years ago (Zotterman, 1939) and were later characterized in a number of mammalian species, including humans (Douglas and Ritchie, 1957; Iggo, 1960; Kumazawa and Perl, 1977; Lynn and Carpenter, 1982; Nordin, 1990; Leem et al., 1993; Vallbo et al., 1993; Fang et al., 2005; for review, see McGlone et al., 2014). The sensory modality subserved by such C fiber low-threshold mechanosensitive receptors (C-LTMRs) long remained enigmatic, but in the last 15 years, psychophysical and functional neuroimaging evidence have emerged indicating that C-LTMRs (commonly called C-tactile fibers in humans; here we will refer to these fibers as C-LTMRs regardless of species) signal pleasant, affective touch in response to slow brushing of the skin (Olausson et al., 2002; Björnsdotter et al., 2009; Löken et al., 2009). C-LTMRs have also been proposed to contribute to mechanical allodynia in rodents and humans (Seal et al., 2009; Nagi et al., 2011), although such a role remains contentious (Liljencrantz et al., 2013; Lou et al., 2013). Notably, activation of C-LTMRs may also have analgesic effects (Delfini et al., 2013; Liljencrantz et al., 2017), possibly through the inhibition of nociceptive C fiber input (Lu and Perl, 2003).

The terminations of several different populations of primary afferent nerve fiber in the superficial dorsal horn have been extensively investigated (Maxwell and Réthelyi, 1987; Todd, 2010). For instance, a major group of non-peptidergic nociceptive C fiber that bind the isolectin B_4_ terminate in the middle third or inner half of lamina II (lamina IIi), where they form central terminals of Type I glomeruli (Ribeiro-da-Silva and Coimbra, 1982; Gerke and Plenderleith, 2004). Type II glomeruli, a group of glomeruli in lamina IIi-III morphologically distinct from Type I glomeruli, is thought to be formed by terminals originating from myelinated A fibers (Réthelyi et al., 1982; Ribeiro-da-Silva and Coimbra, 1982). By contrast, although C-LTMRs are known to terminate in lamina IIi in the mouse (Seal et al., 2009; Li et al., 2011; Abraira et al., 2017), the terminal morphology and synaptology of this class of primary afferent fiber in the dorsal horn remains unknown. Moreover, whereas some interneuronal populations have recently been shown to receive C-LTMR input in the mouse (Abraira et al., 2017), the postsynaptic targets of C-LTMRs remain incompletely characterized. Here we used Vesicular glutamate transporter 3 (VGLUT3) as a marker for C-LTMR terminals, taking advantage of the unique expression of this transporter in C-LTMRs among primary afferent fibers in the adult animal (Seal et al., 2009; Li et al., 2011; Usoskin et al., 2015), to examine the ultrastructure and synaptic organization of these fibers in rat and mouse dorsal horn.

## Materials and Methods

### Animals and tissue preparation

Six adult male Sprague Dawley rats were anaesthetized with sodium pentobarbital (50 mg/kg; i.p.) and transcardially perfused with Phosphate-buffered saline (PBS) (300 mOsm, ∼30 s) followed by PBS containing 4% paraformaldehyde (∼1 l, 30 min). In addition, six adult C57BL/6 mice of either sex were anaesthetized with sodium pentobarbital (200 mg/kg; i.p.) and transcardially perfused with PBS followed by PBS containing 4% paraformaldehyde and (for preembedding and postembedding immunoelectron microscopy; two mice) 1% glutaraldehyde. After perfusion, the spinal cord (thoracic, lumbar, and sacral segments) was removed. Spinal cord pieces were either cryoprotected in 30% sucrose and cut into 40-µm-thick transverse sections on a freezing microtome (for light microscopy) or 100- to 250-µm-thick transverse sections using a Vibratome (for electron microscopy). Sections were stored in anti-freeze solution (30% glycerol and 30% ethylene glycol in PBS) at –20°C until use. All animal experiments were approved by the local Animal Care and Use Committee.

#### Antibodies

Primary antibodies used are outlined in Table 1. One of three antibodies raised in mouse, rabbit or guinea pig was used to detect VGLUT3. To validate their specificity, different combinations of VGLUT3 antibodies were used for double immunofluorescence labeling of spinal cord sections. Furthermore, the rabbit anti-VGLUT3 antibody has been validated for immunohistochemistry using knockout mice (Stensrud et al., 2013). The guinea pig, mouse, and rabbit Vesicular glutamate transporter 1 (VGLUT1) antibodies used here showed identical immunolabeling in the spinal cord and in accordance with previous descriptions in rat and mouse spinal cord using other antibodies (Todd et al., 2003; Alvarez et al., 2004; Persson et al., 2006; Brumovsky et al., 2007). Similarly, guinea pig and rabbit antibodies against Vesicular glutamate transporter 2 (VGLUT2) yielded immunolabeling of the spinal cord in line with previous studies (Todd et al., 2003; Alvarez et al., 2004; Persson et al., 2006; Brumovsky et al., 2007). The Homer1 antibodies used showed a distribution similar to what has been described in the dorsal horn (Gutierrez-Mecinas et al., 2016b), and labeled puncta apposed to VGLUT3^+^ terminals. Importantly, Homer1 immunolabeling has been shown to mark nearly all excitatory synapses in the superficial dorsal horn (Gutierrez-Mecinas et al., 2016b). The antibody against protein kinase Cγ (PKCγ) yielded immunolabeling of cells and processes in the dorsal horn in accordance with previous descriptions (Polgár et al., 1999). Antibodies directed toward calretinin and parvalbumin were previously used in the rat spinal cord (Larsson, 2018) and produced immunolabeling patterns similar to other antibodies (Antal et al., 1990; Ren and Ruda, 1994). The neurokinin 1 receptor (NK1R) antibody produces no labeling in the spinal cord of mice lacking the receptor (Ptak et al., 2002). The tyrosine hydroxylase (TH) antibody yielded immunolabeling of spinal cord as described, including that of a strong innervation of the intermediolateral nucleus (IML; Fuxe et al., 1990; Brumovsky et al., 2006).

**Table 1.** Primary antibodies used in this study

Antigen	Host, isotype	Clone	Immunogen	Supplier	Catalog #	RR_ID	Concentration
Calretinin	Guinea pig	Polyclonal	Mouse protein	Synaptic Systems	214 104	AB_10635160	1:500
Homer1	Rabbit	Polyclonal	Human aa 1–186	Synaptic Systems	160 002	AB_2120990	1:250
Homer1	Rabbit	Polyclonal	Human aa 1–186	Synaptic Systems	160 003	AB_887730	1:250
Neurokinin 1 receptor	Rabbit	Polyclonal	Rat aa 393–407	Sigma	S8305	AB_261562	1:100,000
Parvalbumin	Guinea pig	Polyclonal	Rat protein	Synaptic Systems	195 004	AB_2156476	1:500
Protein kinase Cγ	Guinea pig	Polyclonal	Mouse aa 684–697	Frontier Institute	PKCg-GP-Af350	AB_2571826	1:500
Tyrosine hydroxylase	Rabbit	Polyclonal	Rat protein	Thermo Fisher Scientific	P21962	AB_2539844	1:200
VGLUT1	Guinea pig	Polyclonal	Rat aa 456–560	Synaptic Systems	135 304	AB_887878	1:1000
VGLUT1	Mouse, IgG_2b_	CL2754	Human aa 264–293	Atlas Antibodies	AMAb91041	AB_2665777	1:1000
VGLUT1	Rabbit	Polyclonal	Rat aa 456–460	Synaptic Systems	135 003	AB_2315552	1:2000 (immunogold)
VGLUT2	Guinea pig	Polyclonal	Rat aa 510–582	Synaptic Systems	135 404	AB_887884	1:500
VGLUT2	Rabbit	Polyclonal	Rat aa 510–582	Synaptic Systems	135 402	AB_2187539	1:500
VGLUT3	Guinea pig	Polyclonal	Rat aa 566–588	Frontier Institute	VGLUT3-GP-Af300	AB_2571855	1:100
VGLUT3	Mouse, IgG_2a_	57A8	Mouse aa 583–601	Synaptic Systems	135 211	AB_2636917	1:250–500, 1:1000 (immunoperoxidase)
VGLUT3	Rabbit	Polyclonal	Mouse aa 543–601	Synaptic Systems	135 203	AB_887886	1:500

Concentrations specified are for immunofluorescence unless otherwise noted.

#### Immunofluorescence

Thoracic or lumbar spinal cord were incubated in PBS containing 3% normal goat serum, 0.5% bovine serum albumin (BSA), and 0.5% Triton X-100 (blocking solution), before being incubated in primary antibody solution at room temperature overnight. The primary antibody solution contained mixtures of antibodies as detailed in Table 1. In some experiments, biotinylated isolectin B_4_ (IB_4_) (Life Technologies, catalog #I21414) was used to label non-peptidergic C fiber terminations in the dorsal horn. After rinsing, sections were incubated in a cocktail of secondary antibodies (Table 2) diluted 1:500 in blocking solution. In experiments where biotinylated IB_4_ was used, streptavidin-Alexa Fluor 405 (Life Technologies, catalog #S32351) was added to the secondary antibody solution at a 1:500 dilution. Primary antibodies against NK1R and Homer1 were both raised in rabbit; therefore, to perform NK1R/Homer1/VGLUT3 immunofluorescent labeling, sections were first incubated in NK1R antibody at a low concentration. After incubation in biotinylated goat anti-rabbit secondary antibody (1:200; Vector Laboratories) and streptavidin-horseradish peroxidase (1:100; Life Technologies), the sections were subjected to tyramide signal amplification with Alexa Fluor 568 tyramide (Life Technologies). After this, the sections were subjected to immunolabeling of Homer1 and VGLUT3 using the standard immunofluorescence protocol as above. In all experiments, sections were mounted on slides and coverslipped with Prolong Diamond or SlowFade Diamond (Life Technologies).

**Table 2.** Secondary antibodies used in this study

Host	Target	Conjugate	Supplier	Catalog #	RR_ID	Concentration
Donkey	Rabbit	Brilliant Violet 421	Jackson ImmunoResearch	711-675-152	AB_2651108	1:200
Goat	Guinea pig	Alexa Fluor 568	Thermo Fisher Scientific	A11075	AB_2534119	1:500
Goat	Guinea pig	Alexa Fluor 647	Thermo Fisher Scientific	A21450	AB_141882	1:500
Goat	Rabbit	10 nm gold	British Biocell	EM.GFAR10		1:20
Goat	Mouse	Biotin	Vector Laboratories	BA-9200	AB_2336171	1:400
Goat	Mouse IgG2_a_	Alexa Fluor 488	Thermo Fisher Scientific	A21131	AB_2535771	1:500
Goat	Mouse IgG2_a_	Alexa Fluor 568	Thermo Fisher Scientific	A21134	AB_2535773	1:500
Goat	Mouse IgG2_a_	Alexa Fluor 647	Thermo Fisher Scientific	A21241	AB_2535810	1:500
Goat	Mouse IgG2_b_	Alexa Fluor 647	Thermo Fisher Scientific	A21242	AB_2535811	1:500
Goat	Mouse IgG_3_	Alexa Fluor 488	Thermo Fisher Scientific	A21151	AB_2535784	1:500
Goat	Rabbit	Alexa Fluor 488	Thermo Fisher Scientific	A11034	AB_2576217	1:500
Goat	Rabbit	Alexa Fluor 568	Thermo Fisher Scientific	A11036	AB_10563566	1:500
Goat	Rabbit	Alexa Fluor 647	Thermo Fisher Scientific	A21245	AB_2535813	1:500

### Confocal microscopic analysis

Immunolabeled sections were imaged using a Zeiss LSM700 confocal microscope. For quantitative analysis, z-stacks were obtained of select regions of lamina II throughout the section thickness using a 63×/1.4 oil immersion objective. All image analysis was performed using ImageJ. For analysis of the colocalization of VGLUT3 with VGLUT1, VGLUT2, and IB4, a variant of the optical disector (Polgár et al., 2004) was used to obtain samples of VGLUT3^+^ terminals in lamina IIi. In each of six animals (three rats and three mice), z-stacks of optical slices at 1-µm separation of 113 × 113-µm regions encompassing inner lamina II were obtained in dorsal horns of two lumbar spinal cord sections. The band of VGLUT3^+^ terminals in lamina IIi was outlined and a 50 or 75 µm stretch along this band selected for analysis. An optical slice *n* was chosen as the reference section (based on abundance of VGLUT3^+^ terminals), and the slice *n* + 5 as the lookup section. All VGLUT3^+^ terminals visible in the region of interest in the reference section or the following four sections but not in the lookup section were selected for analysis without reference to the VGLUT1 or VGLUT2 channels. Each terminal was outlined in the slice in which it had the largest cross-sectional area and subsequently assessed for immunoreactivity for VGLUT1 or VGLUT2. In the rat sections, a similar analysis was made with respect to VGLUT3 and VGLUT2 immunolabeling of VGLUT1^+^ terminals in the same regions of interest. to estimate the relative abundance of VGLUT3^+^ and VGLUT1^+^ terminals in lamina IIi in the mouse, the VGLUT3 band was outlined, after which a gray scale lookup table was applied to both the VGLUT3 and VGLUT1 channels, which were subsequently overlaid. In this manner, VGLUT3^+^ and VGLUT1^+^ terminals could not be distinguished from each other in the resulting composite image. After random selection of 50 large (more than ∼1.5 µm in diameter along the longest axis) immunoreactive terminals in each dorsal horn (two dorsal horns in three mice, yielding 300 terminals in total), the channels were alternately switched off to determine whether each terminal was immunoreactive for VGLUT1 or VGLUT3.

For analysis of the size of VGLUT3^+^ terminals and the number of Homer1^+^ puncta associated with such terminals, z-stacks (optical slice separation 0.35 µm, pixel size 42–50 nm) were acquired of sections double immunolabeled for VGLUT3 and Homer1. A 50 × 50-µm region of interest was centered over lamina IIi on the micrograph. As above, the optical disector was used to sample VGLUT3^+^ terminals within this region; here, an optical slice *n* was selected as reference section and *n* + 15 as lookup section. A few terminals were not associated with any discernible Homer1^+^ puncta and were discarded from analysis. Each terminal was outlined in the optical section in which it had its largest cross-sectional area and the maximum Feret diameter measured. Subsequently, all optical slices occupied by the terminal were scanned for apposing Homer1^+^ puncta.

### Preembedding immunoperoxidase labeling

Lumbar or sacral mouse or rat spinal cord sections were used for preembedding immunoperoxidase labeling of VGLUT3. Mouse tissue sections were initially incubated in 1% NaBH_4_ in PBS for 30 min to quench free aldehyde groups. After permeabilization in 50% ethanol for 30 min and incubation in PBS with 1% BSA for 1 h, the sections were incubated in primary antibody solution (mouse anti-VGLUT3, 1:1000 in PBS with 1% BSA) at room temperature overnight or for 72 h. After rinsing, the sections were subsequently incubated in biotinylated goat anti-mouse secondary antibody (Vector Laboratories) in PBS with 1% BSA and in Vector ABC (Vector Laboratories) for 2–3 h each. Peroxidase activity was visualized by incubation in ImmPACT DAB (Vector Laboratories) for 30 s to 2 min. Sections thus labeled for VGLUT3 were rinsed briefly in PB (0.1 M; pH 7.4), incubated in 0.5–1% OsO_4_ in PB for 10–30 min (depending on section thickness), dehydrated in graded series of ethanol and embedded in Durcupan (Electron Microscopy Sciences). Ultrathin sections of embedded tissue were counterstained using 2% uranyl acetate (15 min) or UranyLess (2 min; Electron Microscopy Sciences) followed by 0.4% lead citrate before examination in a JEOL 1230 electron microscope.

### Postembedding immunogold labeling

Vibratome sections of lumbar mouse spinal cord were freeze-substituted and embedded in Lowicryl HM20 Monostep (Electron Microscopy Sciences) as previously described (Larsson et al., 2001). Ultrathin sections (70 nm) collected on single slot Ni grids were subject to VGLUT1 postembedding immunogold labeling. Sections were incubated in Tris-buffered saline [5 mM (pH 7.4) and 0.3% NaCl] with 0.1% Triton X-100 (TBST) and 50 mM glycine to remove free aldehyde groups. After rinsing in TBST and blocking in TBST with 2% human serum albumin (TBST-HSA), sections were incubated in rabbit anti-VGLUT1 (1:2000) in TBST-HSA at room temperature for 2 h. After rinsing, sections were incubated in goat F(ab)_2_ anti-rabbit conjugated to 10 nm gold (British Biocell; Table 2) in TBST-HSA for 1 h. The sections were rinsed in H_2_O, counterstained with uranyl acetate and lead citrate, air dried and examined in the electron microscope.

### Experimental design and statistical analysis

For quantitative analysis of terminal size and number of associated Homer1^+^ puncta, terminals were selected by a stereological technique (see above) from two or three micrographs from two lumbar spinal cord sections each from two rats and mice, respectively (50–59 terminals per animal). Kolmogorov–Smirnov test (terminal size) or two-tailed Mann–Whitney *U* test (Homer1^+^ puncta) were performed using GraphPad Prism 7 to test for differences between species.

## Results

### General distribution of VGLUT3 immunolabeling

**Figure 1.**
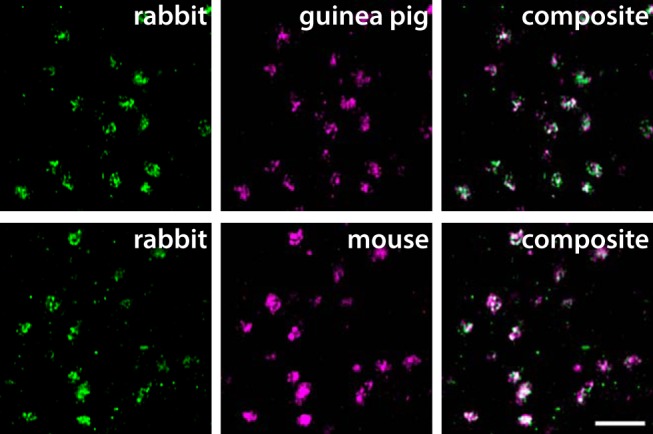
Validation of VGLUT3 antibodies for immunofluorescence. Antibodies raised in rabbit, guinea pig, and mouse label the same terminal-like structures in inner lamina II in the rat spinal cord. The micrographs are single deconvolved optical sections acquired with a 63×/1.4 oil immersion objective. Scale bar, 5 µm, valid for all panels.

Three different antibodies against VGLUT3 were used in this study. To confirm that they all yield specific labeling in mouse and rat spinal cord, we performed double immunofluorescent labeling using different combinations of pairs of VGLUT3 antibodies in rat and mouse spinal cord sections. Indeed, in rat spinal cord, the immunolabeling produced by the antibodies showed essentially identical patterns and near-complete co-localization (Fig. 1), apart from a weak labeling of cell bodies by the rabbit antibody (data not shown). Similar observations were made in the mouse spinal cord except that the guinea pig antibody, which was raised against rat VGLUT3, yielded no immunolabeling.

**Figure 2.**
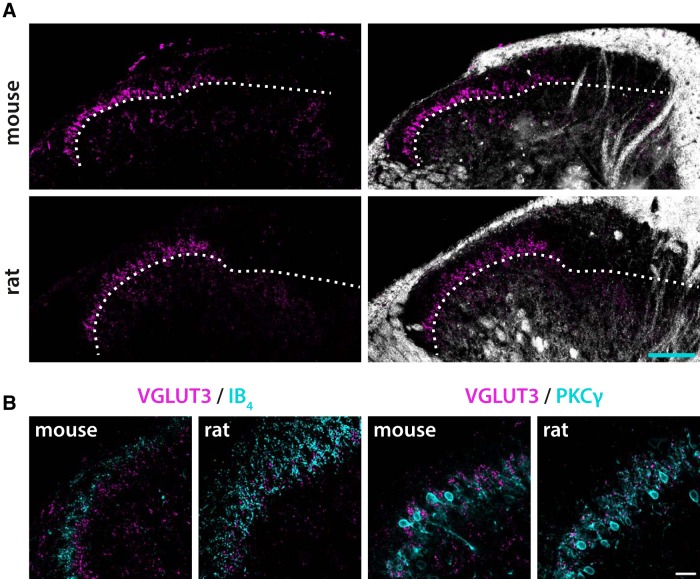
Laminar distribution of VGLUT3 immunolabeling in the rat and mouse dorsal horn. ***A***, VGLUT3 immunofluorescence (magenta) alone (left panels) or superimposed over darkfield micrographs of the labeled sections (right panels). In both mouse and rat spinal cord, a band of VGLUT3^+^ terminal-like structures is evident in the inner part of lamina II. The dashed lines indicate the border between lamina II and III, as assessed from the darkfield micrographs. Note that in these sections, which are from the L4 segment, the band is absent from the medial part of the dorsal horn. The micrographs were obtained with a 10×/0.3 objective. Scalebar, 100 µm, valid for all panels. ***B***, VGLUT3 immunofluorescence relative to IB_4_ binding (left panels) and PKCγ immunolabeling (right panels) in mouse and rat dorsal horn. In mouse dorsal horn, the VGLUT3^+^ band is immediately ventral to the plexus of IB_4_ binding terminals, whereas in the rat, the VGLUT3^+^ band overlaps with the ventral, most intensely labeled part of the IB_4_ band. By contrast, in both mouse and rat dorsal horn, the VGLUT3^+^ band overlaps with the plexus of PKCγ^+^ processes marking lamina IIi. All panels are single optical sections obtained using a 40×/1.3 oil immersion objective. Scale bar, 20 µm, valid for all panels.

The distribution of VGLUT3 immunoreactivity in the mouse superficial dorsal horn was as expected from previous observations (Seal et al., 2009); scattered immunoreactive fibers and puncta were present throughout the superficial laminae, while such fibers were sparser in the deep dorsal horn (Fig. 2*A*). However, the most conspicuous immunolabeling was that of a band of immunoreactive profiles in the ventral part of lamina II. In the rat dorsal horn, a similar plexus of VGLUT3^+^ structures in ventral lamina II was present, although this band showed somewhat weaker immunoreactivity than in the mouse. Furthermore, fewer immunoreactive fibers were present in lamina I, dorsal lamina II, and lamina III in the rat as compared to the mouse. Notably, in both rat and mouse sections from L4 and L5 segments, the band of VGLUT3 immunolabeling in lamina II was restricted to the lateral and intermediate parts of this lamina (Fig. 2*A*), whereas in sections from thoracic, sacral or other lumbar segments the VGLUT3^+^ band extended throughout the mediolateral axis of lamina II (data not shown). This pattern of immunoreactivity is consistent with the projection of C-LTMRs to hairy but not glabrous skin, and is further evidence that the observed VGLUT3 immunolabeling corresponds to the nerve endings of C-LTMRs.

To further characterize the laminar location of VGLUT3^+^ presumed C-LTMR terminations in mouse and rat spinal cord, VGLUT3^+^ immunolabeling was combined with IB_4_ binding or PKCγ immunolabeling, two widely used markers of lamination in the superficial dorsal horn (Silverman and Kruger, 1990; Polgár et al., 1999; Woodbury et al., 2000). In the mouse, the VGLUT3^+^ band was located immediately ventrally to the IB_4_ band and overlapped with the PKCγ plexus (Fig. 2*B*), as previously described (Seal et al., 2009). In rat dorsal horn, however, the VGLUT3^+^ band localized to the ventral part of the IB4 band; the ventral border of the VGLUT3^+^ band closely matched the ventral border of the IB4 band. Closer inspection showed that the VGLUT3^+^ profiles intermingled with IB4^+^ structures, but colocalization was never observed. The VGLUT3^+^ band overlapped with the PKCγ^+^ band, as in the mouse. Comparison with darkfield micrographs suggested that the ventral aspect of the VGLUT3 band coincided with the border between lamina II and III (Fig. 2*A*). Thus, VGLUT3^+^ presumed C-LTMRs terminate in lamina IIi in both rat and mouse spinal cord, but in the rat, IB4^+^ binding C fiber terminals terminate more ventrally than in the mouse and partly intermingle with terminals formed by presumed C-LTMRs.

### TH in C-LTMR terminals

**Figure 3.**
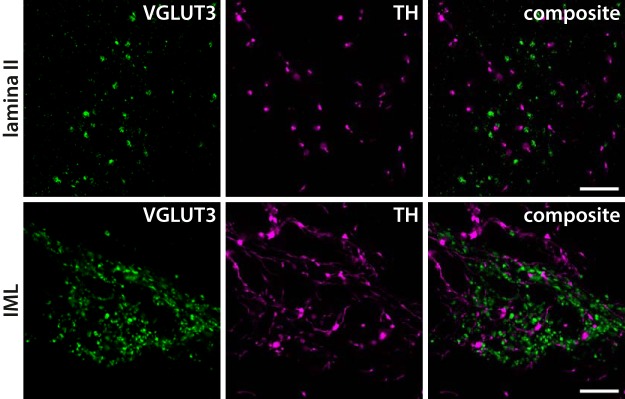
Lack of colocalization of VGLUT3 and TH immunofluorescence in the rat spinal cord. TH immunoreactive processes are found throughout the spinal gray matter, including in lamina II. TH^+^ processes never colocalize with VGLUT3^+^ terminals in lamina II or in the IML in thoracic spinal cord. All panels are single deconvolved optical sections obtained using a 63×/1.4 oil immersion objective. Scale bar, 10 µm, valid for all panels.

C-LTMRs that express VGLUT3 also strongly express TH (Li et al., 2011; Usoskin et al., 2015), suggesting that TH may be a marker of C-LTMR terminals in the spinal cord. However, whether TH is transported to the central terminals of C-LTMRs is not known. To investigate this issue, we used double immunofluorescent labeling of VGLUT3 and TH in spinal cord sections. TH^+^ fibers were scattered throughout the gray matter of the spinal cord. However, in neither rat (Fig. 3) nor mouse (data not shown) dorsal horn did TH colocalize with VGLUT3^+^ terminals, indicating that TH is not present in C-LTMR terminals in the dorsal horn. This is in accordance with previous observations that dorsal rhizotomy does not reduce TH immunoreactivity in the dorsal horn (Brumovsky et al., 2006). In thoracic spinal cord sections, both VGLUT3^+^ and TH^+^ fibers were found in the IML. However, no colocalization were found between VGLUT3^+^ and TH^+^ fibers in this nucleus (Fig. 3).

### Vesicular glutamate transporters in C-LTMR terminals

**Figure 4.**
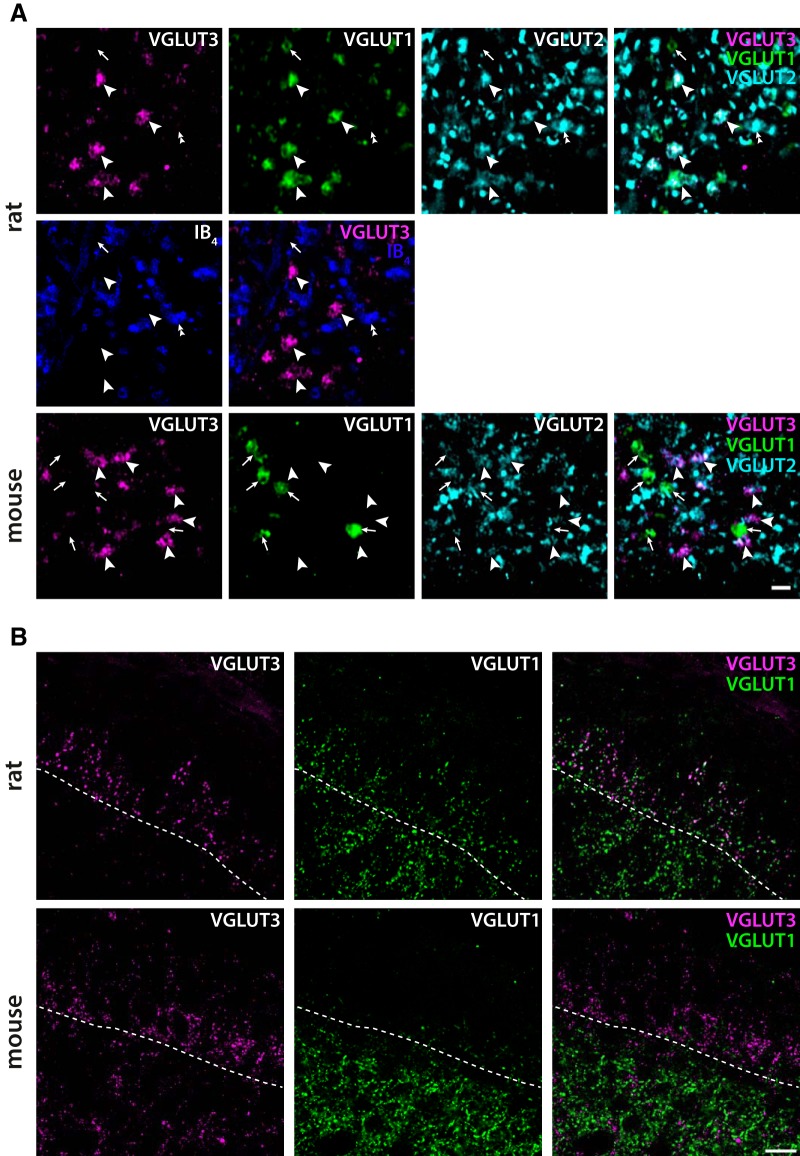
Colocalization of VGLUT3 with VGLUT1 and VGLUT2 in the dorsal horn. ***A***, In the rat spinal cord, VGLUT3^+^ terminals in lamina IIi are also immunoreactive for VGLUT1 and, sometimes weakly, for VGLUT2. VGLUT3^+^ terminals intermingle with IB_4_ binding terminals but never bind IB_4_ themselves. In the mouse, VGLUT3^+^ terminals are not VGLUT1 immunoreactive but exhibit weak-to-moderate VGLUT2 immunolabeling. Arrowheads indicate VGLUT3^+^ terminals, arrows VGLUT1^+^/VGLUT3^-^ terminals, and double arrowhead indicates an IB4^+^ terminal that is VGLUT2 immunoreactive but lacks VGLUT1 or VGLUT3 immunolabeling. The micrographs are single deconvolved optical sections acquired with a 63×/1.4 objective. Scale bar, 1 µm, valid for all panels. ***B***, Overview of VGLUT3^+^ and VGLUT1^+^ immunofluorescence in rat and mouse superficial dorsal horn. VGLUT1^+^ terminals are more abundant in lamina IIi in the rat as compared to the mouse. Note also that most VGLUT1^+^ terminals are VGLUT3^+^ in the rat but not in the mouse. VGLUT3^+^ processes in lamina I, outer lamina II, and in lamina III are more common in the mouse relative to the same laminae in the rat. Dashed lines indicate the border between lamina II and III as judged from the VGLUT3 immunoreactivity. Scale bar, 20 µm, valid for all panels in ***B***.

C-LTMR terminals were reported to not express VGLUT1 or VGLUT2 in the mouse (Seal et al., 2009). Using triple immunofluorescent labeling for VGLUT1, VGLUT2, and VGLUT3, we indeed observed that VGLUT3^+^ terminals never co-localized with VGLUT1 immunoreactivity in mouse spinal cord (Fig. 4; Table 3). By contrast, 80.4 ± 2.6% (mean ± SD; *n* = 3 mice) of VGLUT3^+^ terminals did exhibit VGLUT2 immunoreactivity, although this immunoreactivity in most instances was weak (Fig. 4*A*). Surprisingly, unlike in the mouse, in the rat spinal cord, essentially all (99.4 ± 0.5%; *n* = 3 rats) VGLUT3^+^ terminals showed VGLUT1 immunolabeling (Table 4); this labeling was generally moderate-to-strong. Most (95.1 ± 7.7%) VGLUT3^+^ terminals also showed VGLUT2 immunoreactivity, which ranged from weak to moderate. As VGLUT3^+^ C-LTMR terminals appear to constitute a subset of VGLUT1^+^ terminals in lamina IIi in the rat, we determined the proportion of VGLUT1^+^ terminals in this sublamina that express VGLUT3. In fact, a majority of VGLUT1^+^ terminals (77.9 ± 5.8%; Table 5), were VGLUT3^+^ in lamina IIi. While VGLUT3^+^ presumed C-LTMRs did not express VGLUT1^+^ in the mouse, we assessed the proportions of VGLUT3^+^ and VGLUT1^+^ terminals in the joint set of VGLUT3^+^ terminals and VGLUT1^+^ terminals in lamina IIi. VGLUT3^+^ terminals constituted 78.0 ± 6.2% of this set of terminals (Table 6), thus in close alignment with the proportion of VGLUT3^+^ terminals among VGLUT1^+^ terminals in the rat. Indeed, we noted that substantially fewer VGLUT1^+^ terminals were present in lamina IIi in the mouse compared to the rat (Fig. 4*B*). Furthermore, VGLUT1^+^ terminals were scarce in medial lamina IIi in sections from the rat lumbar enlargement, where VGLUT3^+^ terminals were absent (data not shown).

**Table 3:** Immunolabeling of VGLUT2 and VGLUT3 in VGLUT3^+^ terminals in inner lamina II of mouse dorsal horn

Animal	*n*	% VGLUT1^+^	% VGLUT2^+^
1	117	0 %	81 %
2	80	0 %	78 %
3	86	0 %	83 %

*n*, Number of terminals analyzed in each animal.

**Table 4:** Immunolabeling of VGLUT1 and VGLUT2 in VGLUT3^+^ terminals in inner lamina II of rat dorsal horn

Animal	*n*	% VGLUT1^+^	% VGLUT2^+^	% VGLUT1^+^/VGLUT2^+^
1	117	99 %	99 %	98 %
2	96	100 %	100 %	100 %
3	102	99 %	86 %	85 %

*n*, Number of terminals analyzed in each animal.

**Table 5:** Immunolabeling of VGLUT2 and VGLUT3 in VGLUT1^+^ terminals in inner lamina II of rat dorsal horn

Animal	*n*	% VGLUT2^+^	% VGLUT3^+^	% VGLUT2^+^/VGLUT3^+^
1	106	91 %	85 %	82 %
2	90	83 %	73 %	73 %
3	140	76 %	78 %	71 %

*n*, Number of terminals analyzed in each animal.

**Table 6:** Percentage of VGLUT3^+^ terminals among all VGLUT1^+^ and VGLUT3^+^ terminals in inner lamina II of mouse dorsal horn

Animal	*n*	% VGLUT3^+^
1	100	76 %
2	100	85 %
3	100	73 %

*n*, Number of terminals analyzed in each animal.

### Number of synapses formed by C-LTMR terminals

**Figure 5.**
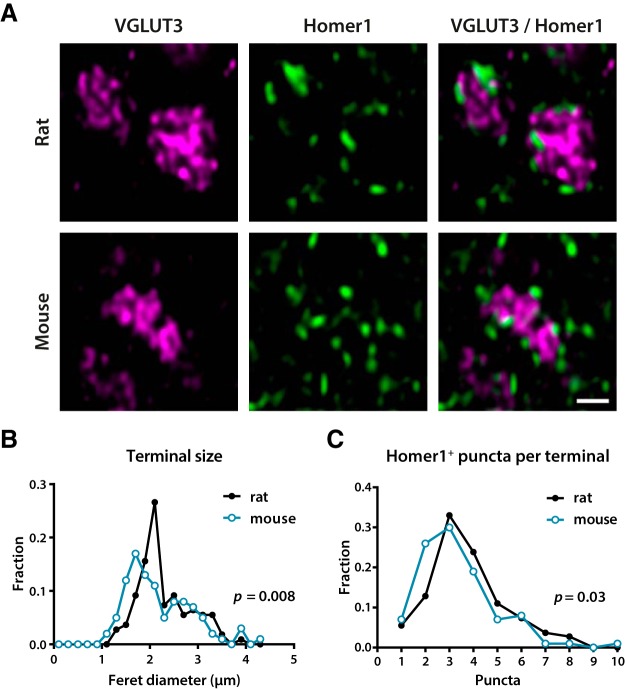
Size and synapse number of VGLUT3^+^ C-LTMRs. ***A***, Examples of VGLUT3^+^ presumed C-LTMRs in lamina IIi in spinal cord sections co-immunolabeled for the excitatory synaptic marker Homer1. Each terminal is apposed to several Homer1^+^ puncta. Note the incomplete filling of the terminals with VGLUT3 immunofluorescence, partly attributed to a high prevalence of axoplasmic mitochondria. The micrographs are single deconvolved optical sections acquired with a 63×/1.4 objective. Scale bar, 1 µm, valid for all panels. ***B***, Frequency distribution of the maximum Feret diameter of VGLUT3^+^ terminals in lamina IIi. Mouse and rat distributions were compared using the Kolmogorov–Smirnov test. ***C***, Histogram of the number of associated Homer1^+^ puncta per VGLUT3^+^ terminal, as assessed from all optical sections occupied by a terminal. Statistical significance of the difference between mouse and rat distributions was tested using Mann–Whitney *U* test.

VGLUT3^+^ terminals in lamina IIi were medium-to-large in size, which may suggest that C-LTMRs form multisynaptic boutons. To investigate this, we performed double immunofluorescent labeling of VGLUT3 and Homer1, a general marker of excitatory synapses in the dorsal horn (Gutierrez-Mecinas et al., 2016b). In transverse sections of rat and mouse spinal cord, VGLUT3^+^ C-LTMR terminals had a median maximum Feret diameter of 2.1 µm (*n* = 109 terminals) and 2.0 µm (*n* = 100 terminals), respectively (Fig. 5). The median number of Homer1^+^ puncta was 3 for both rat and mouse terminals, although some terminals were associated with up to 10 puncta (Fig. 5*C*). Only Homer1^+^ puncta clearly associated with the terminal were included; for instance, puncta situated below or above the terminal in the *z*-axis were excluded. The number of associated Homer1^+^ puncta is therefore likely a lower estimate of the number of excitatory synapses formed by these terminals.

### Ultrastructure of C-LTMR terminals

**Figure 6.**
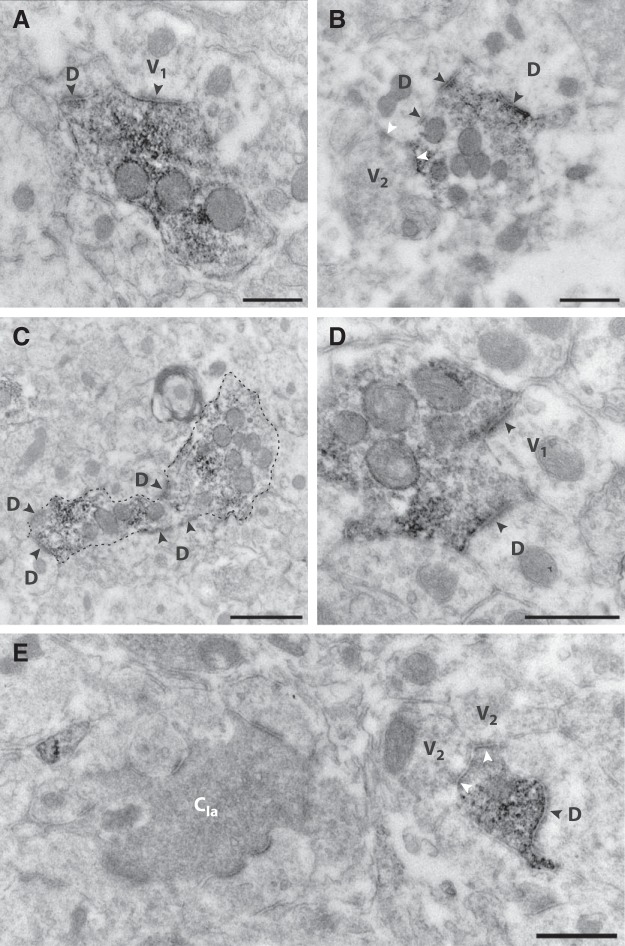
Ultrastructural identification of C-LTMR terminals in the rat. ***A–E***, Examples of terminals showing preembedding immunoperoxidase labeling for VGLUT3 in lamina IIi. Each terminal shows characteristics of central terminals of Type II glomeruli, including a generally round outline, light axoplasm, loosely packed clear small-diameter synaptic vesicles and several mitochondria. The terminals form multiple asymmetric synapses with postsynaptic dendrites, some of which contain vesicles that likely store GABA and thus originate from inhibitory neurons. Peripheral axons form inhibitory symmetric synapses onto either the central terminal (***E***) or a dendrite, or both (***B***). In ***E***, a Type Ia glomeruli is adjacent to a VGLUT3^+^ terminal. Note that the central terminal of the Type I glomeruli (C_Ia_), presumably originating from a non-peptidergic IB_4_ binding C fiber, exhibits no peroxidase labeling. D, postsynaptic dendrite lacking vesicles; V_1_, postsynaptic vesicle-containing dendrite; V_2_, vesicle-containing presynaptic axon. Black and white arrowheads indicate the postsynaptic aspect of asymmetric and symmetric synapses, respectively. Dashed line in ***C*** outlines the terminal for clarity. Scale bars, 500 nm (***A***, ***B***, ***D***, ***E***) and 1 µm (***C***).

The light microscopic observations described above suggest that C-LTMRs may form central terminals of synaptic glomeruli, like many other primary afferent fibers (Maxwell and Réthelyi, 1987; Todd, 2010). We therefore performed preembedding immunoperoxidase labeling and used electron microscopy to examine the ultrastructure of VGLUT3^+^ presumed C-LTMR terminals. In rat dorsal horn, peroxidase-labeled terminals were common in lamina IIi. Many of these could be readily identified as central terminals of Type II glomeruli, as described by Ribeiro-da-Silva and Coimbra (1982), by having light axoplasm, generally loosely packed small and clear vesicles that did not fill the entire axoplasm, at least two asymmetric synapses with postsynaptic dendrites, often several mitochondria, and a relatively round outline (Fig. 6). Neurofilaments were never identified in peroxidase-labeled terminals. Asymmetric synapses were established by labeled terminals with postsynaptic dendrites that lacked vesicles, but also with vesicle-containing dendrites (V_1_ profiles, per the terminology of Ribeiro-da-Silva and Coimbra), most of which likely originate from GABAergic neurons (Todd, 1996). Moreover, peripheral presumed inhibitory V_2_ axons formed symmetric synapses onto the central peroxidase-labeled terminals as well as onto postsynaptic dendrites, thus forming both synaptic dyads and triads (Ribeiro-da-Silva and Coimbra, 1982; Ribeiro-da-Silva et al., 1985) with the central VGLUT3^+^ terminals. As expected, given that each terminal was examined in at most three serial sections, some peroxidase-labeled terminals were relatively small and formed only one synapse in the sections examined. In addition, small immunoreactive vesicle-containing nonsynaptic profiles, presumably preterminal axons, were occasionally observed. Notably, although peroxidase-labeled terminals were intermingled with Type I glomeruli in lamina IIi in the rat dorsal horn, central terminals of Type I glomeruli were never immunoreactive for VGLUT3 (Fig. 6*E*).

**Figure 7.**
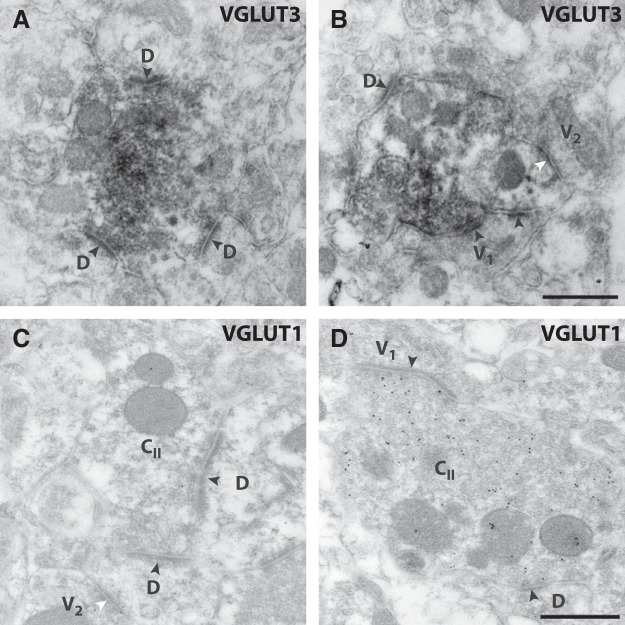
Ultrastructural identification of C-LTMR terminals in the mouse. ***A***, ***B***, Examples of terminals showing preembedding immunoperoxidase labeling for VGLUT3 in lamina IIi of the mouse. As in the rat, peroxidase-labeled terminals were morphologically identical to central terminals of Type II glomeruli. ***C***, ***D***, Lowicryl-embedded dorsal horn section labeled for VGLUT1 using postembedding immunogold labeling. In ***C*,** a central terminal of a Type II glomeruli (C_II_) in lamina IIi devoid of VGLUT1 immunolabeling above background levels is shown. In ***D***, another central terminal of a Type II glomeruli in lamina IIi exhibits strong VGLUT1 immunogold labeling. D, postsynaptic dendrite lacking vesicles; V_1_, postsynaptic vesicle-containing dendrite; V_2_, vesicle-containing presynaptic axon. Black and white arrowheads indicate the postsynaptic aspect of asymmetric and symmetric synapses, respectively. Scale bars, 500 nm (***A***, ***B***) and 500 nm (***C***, ***D***).

In the mouse, VGLUT3^+^ peroxidase-labeled terminals, as in the rat, could often be identified as central terminals of Type II glomeruli (Fig. 7*A*,*B*). In this species, however, such terminals were found ventrally to Type I glomeruli rather than being intermingled with them, in accordance with the location of VGLUT3^+^ terminals ventral to IB_4_ binding sites as observed by fluorescence microscopy (Fig. 4*B*). As VGLUT3^+^ terminals did not exhibit VGLUT1 immunoreactivity in the mouse, we reasoned that many central terminals of Type II glomeruli in lamina IIi, but not those in lamina III, should lack VGLUT1. To test this, we performed postembedding immunogold labeling of VGLUT1 of mouse dorsal horn sections. Indeed, unlike what has been reported for the rat spinal cord (Alvarez et al., 2004), many central terminals of Type II glomeruli in Lamina IIi were devoid of immunogold labeling, or exhibited levels similar to or below those of surrounding tissue (Fig. 7*C*). Central terminals of Type II glomeruli strongly immunogold labeled for VGLUT1 were occasionally found in lamina IIi (Fig. 7*D*), and were common in lamina III.

### Postsynaptic targets of C-LTMRs

**Figure 8.**
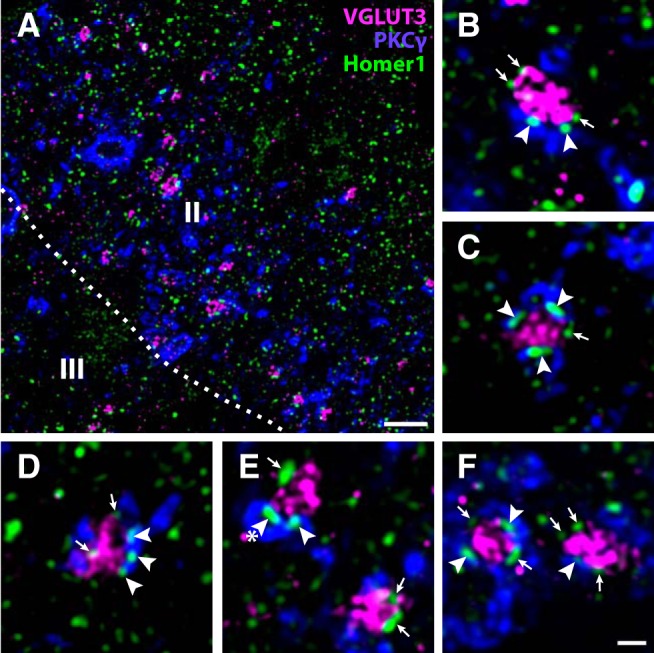
Synaptic connections between VGLUT3^+^ C-LTMRs and PKCγ neurons in rat dorsal horn. ***A***, Overview of a portion of Lamina IIi from a lumbar spinal cord section immunolabeled for VGLUT3, PKCγ, and the excitatory synaptic marker Homer1. Many VGLUT3^+^ terminals are adjacent to PKCγ^+^ dendrites, and often apposed to Homer1^+^ puncta associated with such dendrites. Roman numerals denote Rexed’s laminae. Dashed line indicates border between Lamina II and III. Scale bar, 5 µm. ***B–F***, Examples at higher magnification of VGLUT3^+^ terminals apposed to PKCγ^+^ dendrites. Arrowheads indicate Homer1^+^ puncta associated with PKCγ^+^ dendrites. Arrows indicate Homer1^+^ puncta associated with the VGLUT3^+^ terminals but not with PKCγ^+^ dendrites. Asterisk in e indicates a transversely cut dendritic shaft. Scale bar, 1 µm (***B–F***). All micrographs are single deconvolved optical sections obtained with a 63×/1.4 objective.

Next, we performed triple immunofluorescence labeling of VGLUT3, Homer1 and markers of specific dorsal horn neuronal populations in lumbar rat spinal cord sections to assess whether C-LTMRs establish excitatory synapses with neurons from these populations. C-LTMRs have been shown to provide synaptic input to neurons expressing PKCγ in the mouse (Abraira et al., 2017), but whether such connections are present also in the rat is contentious (Peirs et al., 2014; Andrew and Craig, 2016). Here, we observed that numerous Homer1^+^ puncta in PKCγ^+^ dendrites were associated with VGLUT3^+^ terminals (Fig. 8). Indeed, most VGLUT3^+^ terminals were associated with at least one, and often several, Homer1^+^ puncta in PKCγ dendrites.

**Figure 9.**
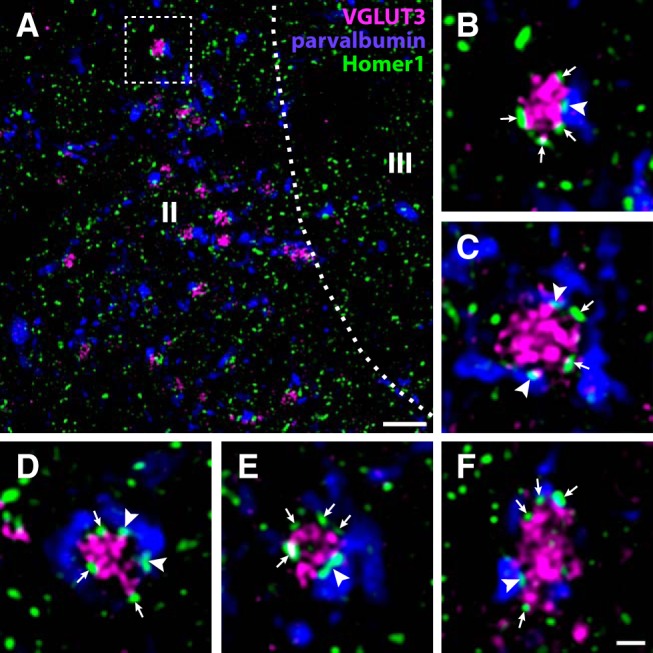
Synaptic connections between VGLUT3^+^ C-LTMRs and parvalbumin neurons in rat dorsal horn. ***A***, A portion of lateral Lamina IIi from a lumbar spinal cord section immunolabeled for VGLUT3, parvalbumin, and Homer1. Many VGLUT3^+^ terminals are apposed to parvalbumin^+^ processes with Homer1^+^ puncta. Roman numerals denote Rexed’s laminae. Dashed line indicates border between lamina II and III. Dashed frame indicates the region magnified in ***B***. Scale bar, 5 µm. ***B–F***, Examples at higher magnification of VGLUT3^+^ terminals apposed to parvalbumin processes. Arrowheads indicate Homer1^+^ puncta, associated with parvalbumin processes, apposed to the VGLUT3^+^ terminals. Arrows indicate Homer1^+^ puncta associated with the VGLUT3^+^ terminals but not with parvalbumin^+^ processes. Scale bar, 1 µm (***B–F***). All micrographs are single deconvolved optical sections obtained with a 63×/1.4 objective.

In sections immunolabeled for parvalbumin together with VGLUT3 and Homer1, we observed that VGLUT3^+^ terminals often apposed parvalbumin^+^ processes, and many of these appositions were associated with Homer1^+^ puncta (Fig. 9). These parvalbumin^+^ processes often showed moderate-to-strong immunoreactivity for parvalbumin; as we have shown that excitatory parvalbumin neurons only weakly express parvalbumin in the rat (Larsson, 2018), this indicates that some of the synapses were onto inhibitory parvalbumin neurons.

**Figure 10.**
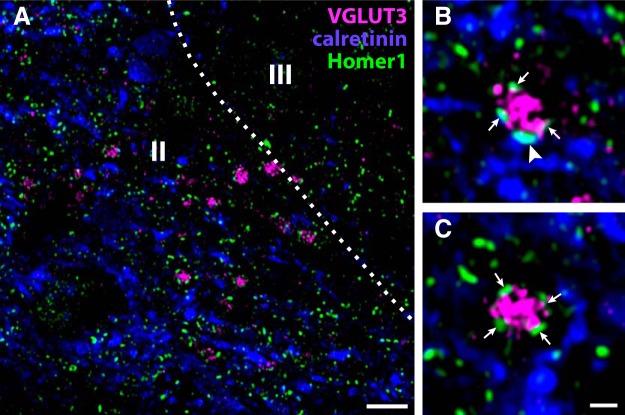
Synaptic connections between VGLUT3^+^ C-LTMRs and calretinin neurons in rat dorsal horn. ***A***, A view of lateral lamina IIi from a lumbar spinal cord section immunolabeled for VGLUT3, calretinin and Homer1. Although calretinin^+^ processes are abundant in lamina IIi, few VGLUT3^+^ terminals are in close proximity to such processes. Roman numerals denote Rexed’s laminae. Dashed line indicates border between lamina II and III. Scale bar, 5 µm. ***B***, A VGLUT3^+^ terminal apposed to a Homer1^+^ puncta associated with a calretinin^+^ process (arrowhead). ***C***, A VGLUT3^+^ terminal apposed to several Homer1^+^ puncta, none of which is associated with the surrounding calretinin^+^ processes. Scale bar, 1 µm (***B***, ***C***). Micrographs in ***A–C*** are deconvolved single optical sections obtained with a 63×/1.4 objective.

Calretinin immunoreactive processes were abundant throughout lamina I and II, including in lamina IIi. However, appositions between VGLUT3^+^ and calretinin^+^ processes were relatively scarce (Fig. 10). Nevertheless, some Homer1^+^ puncta associated with calretinin^+^ processes were juxtaposed to VGLUT3^+^ terminals, indicating a sparse input from C-LTMRs to calretinin^+^ neurons.

**Figure 11.**
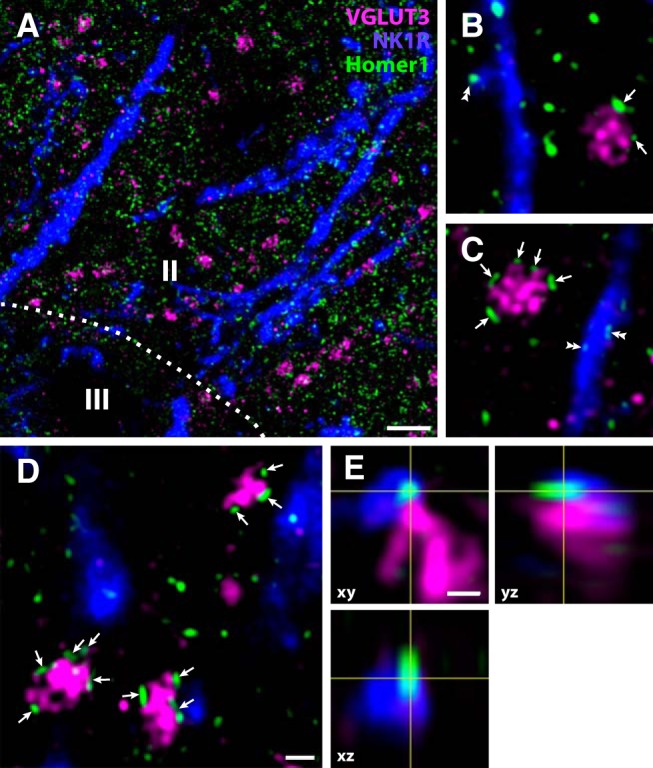
Lack of synaptic connections between VGLUT3^+^ C-LTMRs and NK1R^+^ dendrites in Lamina IIi of the rat dorsal horn. ***A***, A micrograph of an intermediate part of the superficial dorsal horn from a lumbar spinal cord section immunolabeled for VGLUT3, NK1R, and Homer1. Numerous thick and thin NK1R^+^ dendrites traverse Lamina II, but VGLUT3^+^ terminals are rarely juxtaposed to such dendrites. Roman numerals denote Rexed’s laminae. Dashed line indicates border between Lamina II and III. The micrograph is a maximum intensity projection of seven deconvolved optical sections obtained at 0.35-µm separation with a 63×/1.4 objective. Scale bar, 5 µm. ***B–D***, Examples of VGLUT3^+^ terminals in the proximity of, but not apposed to, NK1R^+^ dendrites. Arrows indicate Homer1^+^ puncta apposed to VGLUT3^+^ terminals. Double arrowheads indicate examples of Homer1^+^ puncta associated with NK1R^+^ spines or dendritic shafts. Scale bar, 1 µm (***B–D***). Micrographs are single deconvolved optical sections obtained with a 63×/1.4 objective. ***E***, Orthogonal views of a Homer1^+^ punctum apposed to a VGLUT3^+^ terminal and overlapping with a NK1R^+^ dendritic spine. In the *xy* plane, the Homer1^+^ punctum appears interior to the spine, but sections in the *yz* and *xz* planes shows that the punctum extends outside the spine, indicating that it does not represent a synapse formed by the VGLUT3^+^ terminal on the spine. Scale bar, 500 nm.

While neurons expressing NK1R are rare in lamina II, some NK1R^+^ neurons in laminae I, III, and IV have dendrites that extend into lamina II (Bleazard et al., 1994; Brown et al., 1995; Littlewood et al., 1995), and might therefore conceivably be targets of C-LTMR terminals. In fact, terminals labeled by transganglionically transported cholera toxin B (CTb) have been shown to contact NK1R^+^ dendrites in lamina IIi (Naim et al., 1998), and because C-LTMRs, unlike other C-fibers, transport CTb (Li et al., 2011), some of those terminals might have originated from C-LTMRs. In lumbar spinal cord sections immunolabeled for NK1R, VGLUT3 and Homer1, numerous NK1R^+^ dendrites of varying thickness traversed lamina II, as expected. Some of the dendrites were spiny, and many Homer1^+^ puncta dotted dendritic shafts, in accordance with previous observations of primary afferent synapses onto NK1R^+^ dendrites in lamina II (Naim et al., 1997, 1998). However, VGLUT3^+^ terminals rarely apposed NK1R^+^ dendrites (Fig. 11). In a few instances, Homer1^+^ puncta overlapping with NK1R^+^ dendrites apposed VGLUT3^+^ terminals in a single optical section, but careful examination through the z-stack indicated that such puncta were not confined to the dendrites (Fig. 11*E*). Although we cannot exclude that some of these appositions represented actual synaptic contacts between VGLUT3^+^ terminals and NK1R^+^ dendrites, no unequivocal contacts could be identified in z-stacks encompassing ∼30,000 µm^2^ of lamina IIi.

## Discussion

Here, we have shown that C-LTMR terminals, identified by their expression of VGLUT3, form central terminals of Type II glomeruli in the innermost part of lamina II, where they establish frequent synaptic contacts with PKCγ neurons and parvalbumin neurons, more infrequently contact calretinin neurons and rarely if ever form synapses with NK1R expressing neurons. We have also observed intriguing species differences between rats and mice with respect to coexpression of VGLUT1.

### VGLUT3 as a marker of C-LTMR terminals

In our preparations, three different VGLUT3 antibodies produced weak labeling of a distinct population of terminals in lamina IIi, similar to previous descriptions of the pattern of C-LTMR termination (Seal et al., 2009; Li et al., 2011). Our identification of these terminals as central terminals of synaptic glomeruli confirms their primary afferent origin (Maxwell and Réthelyi, 1987; Ribeiro-da-Silva, 2004; Willis and Coggeshall, 2004). Among primary afferent fibers, only C-LTMRs express VGLUT3 in the mouse (Seal et al., 2009; Li et al., 2011; Usoskin et al., 2015). Given the identical distribution and morphology of VGLUT3^+^ terminals in lamina IIi in the rat as compared to the mouse, it appears highly likely that C-LTMRs exhibit a similar selective expression of VGLUT3 also in the rat. Thus, the medium-to-large diameter VGLUT3^+^ terminals in lamina IIi can be concluded to originate from C-LTMRs in either species. Notably, however, scattered VGLUT3^+^ processes and terminal-like profiles were found throughout the gray matter, and the origins of those remain to be elucidated.

### VGLUTs in C-LTMR terminals

As previously reported (Seal et al., 2009), we found that C-LTMRs did not express VGLUT1 in the mouse. Surprisingly, however, in the rat, these terminals exhibited strong VGLUT1 expression. In addition, weak-to-moderate VGLUT2 expression was found in the majority of C-LTMRs in the rat, and, generally more weakly, in the mouse. Low levels of VGLUT2 protein in mouse C-LTMR terminals and the use of different antibodies may contribute to the discrepancy with the previous study by Seal and co-workers, who did not find VGLUT2 in such terminals (Seal et al., 2009). Notably, a transcriptomic study found that mouse C-LTMRs express the *Slc17a6* gene that encodes VGLUT2 (Usoskin et al., 2015), supporting the present observations. Although the expression of VGLUTs is mostly segregated, co-expression of VGLUT1 and VGLUT2 has been reported (Boulland et al., 2004; Herzog et al., 2006), including in the dorsal horn (Todd et al., 2003; Alvarez et al., 2004; Persson et al., 2006; Brumovsky et al., 2007). VGLUT3 is generally not co-expressed with other VGLUTs but is instead usually associated with non-glutamatergic neurons or glia (Fremeau et al., 2002; Gras et al., 2002; Schäfer et al., 2002; Boulland et al., 2004; Stensrud et al., 2013). Rat C-LTMRs to our knowledge constitute the first reported neuronal population that expresses all three VGLUTs.

The functional consequence of the co-expression of multiple VGLUTs in the same terminal is unclear. Whereas transport kinetics are similar between isoforms, VGLUT1 is associated with lower release probability and short-term depression compared to VGLUT2 and VGLUT3, by virtue of its unique binding to endophilin A1 (Weston et al., 2011). Thus, the differential VGLUT1 expression in rat and mouse C-LTMRs observed here may indicate species differences in release probability and short-term plasticity at C-LTMR synapses.

### Morphology of C-LTMR terminals

By confocal microscopy, we observed that VGLUT3^+^ C-LTMRs form medium-to-large-sized terminals each establishing multiple synaptic connections with postsynaptic neurons, indicating a glomerular organization of such terminals. Preembedding immunoperoxidase labeling and electron microscopy confirmed that C-LTMRs form central terminals of synaptic glomeruli, like many other primary afferent fibers (Maxwell and Réthelyi, 1987; Ribeiro-da-Silva, 2004; Willis and Coggeshall, 2004). Two major types of glomeruli have been identified in the rat spinal cord: Type I glomeruli, associated with non-peptidergic nociceptive C fibers, and Type II glomeruli, which are attributed to myelinated A fibers (Ribeiro-da-Silva and Coimbra, 1982; Ribeiro-da-Silva et al., 1985). Similar synaptic glomeruli are found in primates (Knyihar-Csillik et al., 1982), and the synaptic integration afforded by such complexes is thus likely a general characteristic of mammalian somatosensory signaling. Type II glomeruli were further divided into Type IIa and IIb; the latter have central terminals containing neurofilaments, presumed to correspond to thick myelinated Aβ fibers, whereas Type IIa glomeruli without neurofilaments are thought to originate from thin Aδ fibers (Ribeiro-da-Silva, 2004). Of these, Type IIa glomeruli predominate in lamina IIi. Here we found that C-LTMRs establish central terminals of Type IIa glomeruli. Remarkably, the ratio of VGLUT3^+^ terminals to those that only express VGLUT1 in lamina IIi of both mouse and rat was ∼4:1, identical to the ratio of Type IIa to Type IIb glomeruli in this region (Ribeiro-da-Silva and Coimbra, 1982). This is indeed consistent with the selective lack of neurofilament proteins in C fibers, including C-LTMRs (Usoskin et al., 2015). Thus, we propose that most, if not all, Type IIa glomeruli in lamina IIi associate with C-LTMRs rather than Aδ fibers, whereas myelinated A fibers form Type IIb glomeruli. Furthermore, lack of VGLUT1 expression can be used in the mouse (but not in the rat) to conclusively differentiate C-LTMR terminals from A fiber terminals.

### Synaptic partners of C-LTMRs

C-LTMRs were found to form presumed synaptic connections with parvalbumin neurons in the rat, in agreement with recent observations (Abraira et al., 2017). Whereas we did not directly assess whether the postsynaptic parvalbumin neurons were inhibitory or excitatory, we have recently shown that excitatory parvalbumin neurons invariably exhibit weak parvalbumin immunoreactivity (Larsson, 2018). Many parvalbumin^+^ dendrites receiving contacts from VGLUT3^+^ terminals were strongly parvalbumin immunoreactive, and these therefore likely originate from inhibitory neurons. This contrasts with recent observations in the mouse, where parvalbumin^+^ cells postsynaptic to C-LTMRs were judged, based on morphology, to be excitatory (Abraira et al., 2017). Further studies using selective markers to differentiate between inhibitory and excitatory parvalbumin neurons are needed to assess whether this discrepancy is attributed to species differences.

While neurons possessing NK1R are rare in lamina II, NK1R^+^ neurons in adjacent laminae, including laminae III-IV but also, less prominently, lamina I, have dendrites that extend into this lamina in the rat spinal cord (Bleazard et al., 1994; Brown et al., 1995; Littlewood et al., 1995). Thus, primary afferent fibers that terminate exclusively in lamina II could still contact NK1R^+^ dendrites from neurons in laminae I, III, and IV. Indeed, some lamina III-IV neurons with dendrites in lamina II receive input from nociceptive fibers (Naim et al., 1997). However, the present results showing few if any synaptic connections between VGLUT3^+^ terminals and NK1R^+^ dendrites argue against direct synaptic input to locally or supraspinally projecting NK1R^+^ neurons. Nevertheless, some projection neurons in laminae I, III, and IV lack the NK1R (Todd et al., 2000; Cameron et al., 2015), and some of these could conceivably receive monosynaptic input from C-LTMRs.

The present observations of a considerable synaptic input from VGLUT3^+^ C-LTMRs to PKCγ^+^ neurons are in line with a previous study in the mouse (Abraira et al., 2017). PKCγ^+^ neurons have recently been shown to exhibit considerable heterogeneity with respect to neuropeptide expression (Gutierrez-Mecinas et al., 2016a). Whether different subpopulations of PKCγ^+^ neurons show differential connectivity with C-LTMRs and other primary afferent classes is a topic for further study.

We found restricted synaptic connections between VGLUT3^+^ terminals and calretinin^+^ neurons. As the calretinin^+^ population of neurons shows considerable heterogeneity with respect to transmitter phenotype, morphology and electrophysiology (Smith et al., 2015; Larsson, 2018), it is possible that certain subpopulations of calretinin^+^ neuron are preferentially innervated by C-LTMRs.

### Functional considerations

That C-LTMRs participate in synaptic glomeruli where both the central terminal and postsynaptic dendrites are subject to inhibition, indicates that C-LTMR-mediated signaling is locally regulated at the first synapse, including via primary afferent depolarization (PAD). One role of PAD in cutaneous afferent fibers has been proposed to be to increase spatial discrimination by lateral inhibition (Rudomin, 2009), but given the extremely poor spatial resolution of C fiber-mediated touch (McGlone et al., 2014), this appears unlikely to be a major function for PAD in the case of C-LTMR signaling. Another possible role for PAD may be to prevent hyperactivity of low-threshold afferent systems (Orefice et al., 2016).

The synaptic connections of C-LTMR terminals revealed here and elsewhere (Abraira et al., 2017) indicate that activation of C-LTMRs engage both excitatory and inhibitory pathways in the dorsal horn. C-LTMR-mediated inhibition of nociceptive signaling could tentatively provide a basis for slow brush-mediated analgesia (Liljencrantz et al., 2017); conversely, disruption of such inhibition may contribute to tactile allodynia. Moreover, the extensive synaptic connections between C-LTMRs and PKCγ neurons, which have been implicated in mechanical allodynia (Todd, 2017), indicate a potential mechanism by which C-LTMR activation could lead to pain under certain conditions.

Wide dynamic range lamina I neurons that project to the lateral parabrachial nucleus are activated by slow brushing, and thus likely receive C-LTMR input (Andrew, 2010). The present observations are in alignment with previous electrophysiological evidence (Andrew, 2010) that such input is not monosynaptic but is instead relayed via interneurons. One class of interneurons which may have such a role is the PKCγ^+^ population (Andrew and Craig, 2016). Intriguingly, a morphologically distinct class of excitatory interneuron known as vertical cells has been shown in rats to receive contacts in lamina IIi from VGLUT1^+^ primary afferent fibers that transport CTb (Yasaka et al., 2014). As C-LTMRs express both VGLUT1 (in the rat; present study) and transport CTb (Li et al., 2011), some of these contacts may be from C-LTMRs rather than from A fibers. Indeed, vertical cells may be activated by slow brushing (Light et al., 1979). Because vertical cells establish synapses with spinoparabrachial lamina I neurons (Cordero-Erausquin et al., 2009), these cells could be one possible route by which slow brushing and C-LTMRs activate lamina I projection neurons.

## Synthesis

Reviewing Editor: Mary Heinricher, Oregon Health & Science University Decisions are customarily a result of the Reviewing Editor and the peer reviewers coming together and discussing their recommendations until a consensus is reached. When revisions are invited, a fact-based synthesis statement explaining their decision and outlining what is needed to prepare a revision will be listed below. The following reviewer(s) agreed to reveal their identity: NONE. Note: If this manuscript was transferred from JNeurosci and a decision was made to accept the manuscript without peer review, a brief statement to this effect will instead be what is listed below.

The manuscript was accepted after editorial review.

## References

[B1] Abraira VE, Kuehn ED, Chirila AM, Springel MW, Toliver AA, Zimmerman AL, Orefice LL, Boyle KA, Bai L, Song BJ, Bashista KA, O'Neill TG, Zhuo J, Tsan C, Hoynoski J, Rutlin M, Kus L, Niederkofler V, Watanabe M, Dymecki SM, et al. (2017) The cellular and synaptic architecture of the mechanosensory dorsal horn. Cell 168:295–310.e19. 10.1016/j.cell.2016.12.010 28041852 PMC5236062

[B2] Alvarez FJ, Villalba RM, Zerda R, Schneider SP (2004) Vesicular glutamate transporters in the spinal cord, with special reference to sensory primary afferent synapses. J Comp Neurol 472:257–280. 10.1002/cne.20012 15065123

[B3] Andrew D (2010) Quantitative characterization of low-threshold mechanoreceptor inputs to lamina I spinoparabrachial neurons in the rat. J Physiol 588:117–124. 10.1113/jphysiol.2009.181511 19933757 PMC2821553

[B4] Andrew D, Craig AD (2016) Processing of C-tactile information in the spinal cord In: Affective touch and the neurophysiology of CT afferents (Olausson H, Wessberg J, Morrison I, McGlone F, eds), pp 159–173. New York: Springer.

[B5] Antal M, Freund TF, Polgár E (1990) Calcium-binding proteins, parvalbumin- and calbindin-D 28k-immunoreactive neurons in the rat spinal cord and dorsal root ganglia: a light and electron microscopic study. J Comp Neurol 295:467–484. 10.1002/cne.902950310 2351764

[B6] Björnsdotter M, Löken L, Olausson H, Vallbo Å, Wessberg J (2009) Somatotopic organization of gentle touch processing in the posterior insular cortex. J Neurosci 29:9314–9320. 10.1523/JNEUROSCI.0400-09.2009 19625521 PMC6665561

[B7] Bleazard L, Hill R, Morris R (1994) The correlation between the distribution of the NK1 receptor and the actions of tachykinin agonists in the dorsal horn of the rat indicates that substance P does not have a functional role on substantia gelatinosa (lamina II) neurons. J Neurosci 14:7655–7664. 10.1523/JNEUROSCI.14-12-07655.1994 7527847 PMC6576917

[B8] Boulland J-L, Qureshi T, Seal RP, Rafiki A, Gundersen V, Bergersen LH, Fremeau RT, Edwards RH, Storm-Mathisen J, Chaudhry FA (2004) Expression of the vesicular glutamate transporters during development indicates the widespread corelease of multiple neurotransmitters. J Comp Neurol 480:264–280. 10.1002/cne.20354 15515175

[B9] Brown JL, Liu H, Maggio JE, Vigna SR, Mantyh PW, Basbaum AI (1995) Morphological characterization of substance P receptor-immunoreactive neurons in the rat spinal cord and trigeminal nucleus caudalis. J Comp Neurol 356:327–344. 10.1002/cne.903560302 7642798

[B10] Brumovsky P, Villar MJ, Hökfelt T (2006) Tyrosine hydroxylase is expressed in a subpopulation of small dorsal root ganglion neurons in the adult mouse. Exp Neurol 200:153–165. 10.1016/j.expneurol.2006.01.023 16516890

[B11] Brumovsky P, Watanabe M, Hökfelt T (2007) Expression of the vesicular glutamate transporters-1 and -2 in adult mouse dorsal root ganglia and spinal cord and their regulation by nerve injury. Neuroscience 147:469–490. 10.1016/j.neuroscience.2007.02.068 17577523

[B12] Cameron D, Polgár E, Gutierrez-Mecinas M, Gomez-Lima M, Watanabe M, Todd AJ (2015) The organisation of spinoparabrachial neurons in the mouse. Pain 156:2061–2071. 10.1097/j.pain.0000000000000270 26101837 PMC4770364

[B13] Cordero-Erausquin M, Allard S, Dolique T, Bachand K, Ribeiro-da-Silva A, De Koninck Y (2009) Dorsal horn neurons presynaptic to lamina I spinoparabrachial neurons revealed by transynaptic labeling. J Comp Neurol 517:601–615. 10.1002/cne.22179 19824098

[B14] Delfini M-C, Mantilleri A, Gaillard S, Hao J, Reynders A, Malapert P, Alonso S, François A, Barrere C, Seal R, Landry M, Eschallier A, Alloui A, Bourinet E, Delmas P, Le Feuvre Y, Moqrich A (2013) TAFA4, a chemokine-like protein, modulates injury-induced mechanical and chemical pain hypersensitivity in mice. Cell Rep 5:378–388. 10.1016/j.celrep.2013.09.013 24139797

[B15] Douglas WW, Ritchie JM (1957) Non-medullated fibres in the saphenous nerve which signal touch. J Physiol 139:385–399. 10.1113/jphysiol.1957.sp005899 13492231 PMC1358739

[B16] Fang X, McMullan S, Lawson SN, Djouhri L (2005) Electrophysiological differences between nociceptive and non-nociceptive dorsal root ganglion neurones in the rat in vivo. J Physiol 565:927–943. 10.1113/jphysiol.2005.086199 15831536 PMC1464557

[B17] Fremeau RT, Burman J, Qureshi T, Tran CH, Proctor J, Johnson J, Zhang H, Sulzer D, Copenhagen DR, Storm-Mathisen J, Reimer RJ, Chaudhry FA, Edwards RH (2002) The identification of vesicular glutamate transporter 3 suggests novel modes of signaling by glutamate. Proc Natl Acad Sci USA 99:14488–14493. 10.1073/pnas.222546799 12388773 PMC137910

[B18] Fuxe K, Tinner B, Bjelke B, Agnati LF, Verhofstad A, Steinbusch HGW, Goldstein M, Kalia M (1990) Monoaminergic and peptidergic innervation of the intermedio-lateral horn of the spinal cord. Eur J Neurosci 2:430–450. 10.1111/j.1460-9568.1990.tb00435.x 12106030

[B19] Gerke MB, Plenderleith MB (2004) Ultrastructural analysis of the central terminals of primary sensory neurones labelled by transganglionic transport of bandeiraea simplicifolia i-isolectin B4. Neuroscience 127:165–175. 10.1016/j.neuroscience.2004.05.008 15219679

[B20] Gras C, Herzog E, Bellenchi GC, Bernard V, Ravassard P, Pohl M, Gasnier B, Giros B, El Mestikawy S (2002) A third vesicular glutamate transporter expressed by cholinergic and serotoninergic neurons. J Neurosci 22:5442–5451. 10.1523/jneurosci.22-13-05442.2002 12097496 PMC6758212

[B21] Gutierrez-Mecinas M, Furuta T, Watanabe M, Aj T (2016a) A quantitative study of neurochemically defined excitatory interneuron populations in laminae I-III of the mouse spinal cord. Mol Pain 12:1744806916629065. 10.1177/1744806916629065 27030714 PMC4946630

[B22] Gutierrez-Mecinas M, Kuehn ED, Abraira VE, Polgár E, Watanabe M, Todd AJ (2016b) Immunostaining for Homer reveals the majority of excitatory synapses in laminae I-III of the mouse spinal dorsal horn. Neuroscience 329:171–181. 10.1016/j.neuroscience.2016.05.009 27185486 PMC4915440

[B23] Herzog E, Takamori S, Jahn R, Brose N, Wojcik SM (2006) Synaptic and vesicular co-localization of the glutamate transporters VGLUT1 and VGLUT2 in the mouse hippocampus. J Neurochem 99:1011–1018. 10.1111/j.1471-4159.2006.04144.x 16942593

[B24] Iggo A (1960) Cutaneous mechanoreceptors with afferent C fibres. J Physiol 152:337–353. 10.1113/jphysiol.1960.sp006491 13852622 PMC1363319

[B25] Knyihar-Csillik E, Csillik B, Rakic P (1982) Ultrastructure of normal and degenerating glomerular terminals of dorsal root axons in the substantia gelatinosa of the rhesus monkey. J Comp Neurol 210:357–375. 10.1002/cne.902100404 7142447

[B26] Kumazawa T, Perl ER (1977) Primate cutaneous sensory units with unmyelinated (C) afferent fibers. J Neurophysiol 40:1325–1338. 10.1152/jn.1977.40.6.1325 411895

[B27] Larsson M (2018) Non-canonical heterogeneous cellular distribution and co-localization of CaMKIIα and CaMKIIβ in the spinal superficial dorsal horn. Brain Struct Funct 223:1437–1457. 10.1007/s00429-017-1566-0 29151114 PMC5869946

[B28] Larsson M, Persson S, Ottersen OP, Broman J (2001) Quantitative analysis of immunogold labeling indicates low levels and non-vesicular localization of L-aspartate in rat primary afferent terminals. J Comp Neurol 430:147–159. 10.1002/1096-9861(20010205)430:2<147::AID-CNE1021>3.0.CO;2-5 11135252

[B29] Leem JW, Willis WD, Chung JM (1993) Cutaneous sensory receptors in the rat foot. J Neurophysiol 69:1684–1699. 10.1152/jn.1993.69.5.1684 8509832

[B30] Li L, Rutlin M, Abraira VE, Cassidy C, Kus L, Gong S, Jankowski MP, Luo W, Heintz N, Koerber HR, Woodbury CJ, Ginty DD (2011) The functional organization of cutaneous low-threshold mechanosensory neurons. Cell 147:1615–1627. 10.1016/j.cell.2011.11.027 22196735 PMC3262167

[B31] Light AR, Trevino DL, Perl ER (1979) Morphological features of functionally defined neurons in the marginal zone and substantia gelatinosa of the spinal dorsal horn. J Comp Neurol 186:151–171. 10.1002/cne.901860204 447881

[B32] Liljencrantz J, Björnsdotter M, Morrison I, Bergstrand S, Ceko M, Seminowicz DA, Cole J, Bushnell CM, Olausson H (2013) Altered C-tactile processing in human dynamic tactile allodynia. Pain 154:227–234. 10.1016/j.pain.2012.10.024 23290550

[B33] Liljencrantz J, Strigo I, Ellingsen DM, Krämer HH, Lundblad LC, Nagi SS, Leknes S, Olausson H (2017) Slow brushing reduces heat pain in humans. Eur J Pain 21:1173–1185. 10.1002/ejp.1018 28263013

[B34] Littlewood NK, Todd AJ, Spike RC, Watt C, Shehab SAS (1995) The types of neuron in spinal dorsal horn which possess neurokinin-1 receptors. Neuroscience 66:597–608. 10.1016/0306-4522(95)00039-L 7543982

[B35] Lou S, Duan B, Vong L, Lowell BB, Ma Q (2013) Runx1 controls terminal morphology and mechanosensitivity of VGLUT3-expressing C-mechanoreceptors. J Neurosci 33:870–882. 10.1523/JNEUROSCI.3942-12.2013 23325226 PMC3652638

[B36] Lu Y, Perl ER (2003) A specific inhibitory pathway between substantia gelatinosa neurons receiving direct C-fiber input. J Neurosci 23:8752–8758. 10.1523/jneurosci.23-25-08752.2003 14507975 PMC6740424

[B37] Lynn B, Carpenter SE (1982) Primary afferent units from the hairy skin of the rat hind limb. Brain Res 238:29–43. 10.1016/0006-8993(82)90768-5 6282398

[B38] Löken LS, Wessberg J, Morrison I, McGlone F, Olausson H (2009) Coding of pleasant touch by unmyelinated afferents in humans. Nat Neurosci 12:547–548. 10.1038/nn.2312 19363489

[B39] Maxwell DJ, Réthelyi M (1987) Ultrastructure and synaptic connections of cutaneous afferent fibres in the spinal cord. Trends Neurosci 10:117–123. 10.1016/0166-2236(87)90056-7

[B40] McGlone F, Wessberg J, Olausson H (2014) Discriminative and affective touch: sensing and feeling. Neuron 82:737–755. 10.1016/j.neuron.2014.05.001 24853935

[B41] Nagi SS, Rubin TK, Chelvanayagam DK, Macefield VG, Mahns DA (2011) Allodynia mediated by C-tactile afferents in human hairy skin. J Physiol 589:4065–4075. 10.1113/jphysiol.2011.211326 21727219 PMC3180003

[B42] Naim M, Spike RC, Watt C, Shehab SA, Todd AJ (1997) Cells in laminae III and IV of the rat spinal cord that possess the neurokinin-1 receptor and have dorsally directed dendrites receive a major synaptic input from tachykinin-containing primary afferents. J Neurosci 17:5536–5548. 10.1523/JNEUROSCI.17-14-05536.1997 9204935 PMC6793839

[B43] Naim MM, Shehab SA, Todd AJ (1998) Cells in laminae III and IV of the rat spinal cord which possess the neurokinin-1 receptor receive monosynaptic input from myelinated primary afferents. Eur J Neurosci 10:3012–3019. 10.1111/j.1460-9568.1998.00335.x 9758171

[B44] Nordin M (1990) Low-threshold mechanoreceptive and nociceptive units with unmyelinated (C) fibres in the human supraorbital nerve. J Physiol 426:229–240. 10.1113/jphysiol.1990.sp018135 2231398 PMC1189885

[B45] Olausson H, Lamarre Y, Backlund H, Morin C, Wallin BG, Starck G, Ekholm S, Strigo I, Worsley K, Vallbo AB, Bushnell MC (2002) Unmyelinated tactile afferents signal touch and project to insular cortex. Nat Neurosci 5:900–904. 10.1038/nn896 12145636

[B46] Orefice LL, Zimmerman AL, Chirila AM, Sleboda SJ, Head JP, Ginty DD (2016) Peripheral mechanosensory neuron dysfunction underlies tactile and behavioral deficits in mouse models of ASDs. Cell 166:299–313. 10.1016/j.cell.2016.05.033 27293187 PMC5567792

[B47] Peirs C, Patil S, Bouali-Benazzouz R, Artola A, Landry M, Dallel R (2014) Protein kinase C gamma interneurons in the rat medullary dorsal horn: distribution and synaptic inputs to these neurons, and subcellular localization of the enzyme. J Comp Neurol 522:393–413. 10.1002/cne.23407 23818225

[B48] Persson S, Boulland J-L, Aspling M, Larsson M, Fremeau RTJ, Edwards RH, Storm-Mathisen J, Chaudhry FA, Broman J (2006) Distribution of vesicular glutamate transporters 1 and 2 in the rat spinal cord, with a note on the spinocervical tract. J Comp Neurol 497:683–701. 10.1002/cne.20987 16786558

[B49] Polgár E, Fowler JH, McGill MM, Todd AJ (1999) The types of neuron which contain protein kinase C gamma in rat spinal cord. Brain Res 833:71–80. 10.1016/s0006-8993(99)01500-0 10375678

[B50] Polgár E, Gray S, Riddell JS, Todd AJ (2004) Lack of evidence for significant neuronal loss in laminae I–III of the spinal dorsal horn of the rat in the chronic constriction injury model. Pain 111:144–150. 10.1016/j.pain.2004.06.011 15327818

[B51] Ptak K, Burnet H, Blanchi B, Sieweke M, De Felipe C, Hunt SP, Monteau R, Hilaire G (2002) The murine neurokinin NK1 receptor gene contributes to the adult hypoxic facilitation of ventilation. Eur J Neurosci 16:2245–2252. 10.1046/j.1460-9568.2002.02305.x 12492418

[B52] Ren K, Ruda MA (1994) A comparative study of the calcium-binding proteins calbindin-D28K, calretinin, calmodulin and parvalbumin in the rat spinal cord. Brain Res Brain Res Rev 19:163–179. 10.1016/0165-0173(94)90010-8 8061685

[B53] Réthelyi M, Light AR, Perl ER (1982) Synaptic complexes formed by functionally defined primary afferent units with fine myelinated fibers. J Comp Neurol 207:381–393. 10.1002/cne.902070409 6288776

[B54] Ribeiro-da-Silva A (2004) Substantia gelatinosa of the spinal cord In: The rat nervous system (Paxinos G, ed), Ed 3, pp 129–148. San Diego: Academic Press.

[B55] Ribeiro-da-Silva A, Coimbra A (1982) Two types of synaptic glomeruli and their distribution in laminae I-III of the rat spinal cord. J Comp Neurol 209:176–186. 10.1002/cne.902090205 6890076

[B56] Ribeiro-da-Silva A, Pignatelli D, Coimbra A (1985) Synaptic architecture of glomeruli in superficial dorsal horn of rat spinal cord, as shown in serial reconstructions. J Neurocytol 14:203–220. 10.1007/bf01258448 4045504

[B57] Rudomin P (2009) In search of lost presynaptic inhibition. Exp Brain Res 196:139–151. 10.1007/s00221-009-1758-9 19322562

[B58] Schäfer MK-H, Varoqui H, Defamie N, Weihe E, Erickson JD (2002) Molecular cloning and functional identification of mouse vesicular glutamate transporter 3 and its expression in subsets of novel excitatory neurons. J Biol Chem 277:50734–50748. 10.1074/jbc.M206738200 12384506

[B59] Seal RP, Wang X, Guan Y, Raja SN, Woodbury CJ, Basbaum AI, Edwards RH (2009) Injury-induced mechanical hypersensitivity requires C-low threshold mechanoreceptors. Nature 462:651–655. 10.1038/nature08505 19915548 PMC2810205

[B60] Silverman JD, Kruger L (1990) Selective neuronal glycoconjugate expression in sensory and autonomic ganglia: relation of lectin reactivity to peptide and enzyme markers. J Neurocytol 19:789–801. 10.1007/BF01188046 2077115

[B61] Smith KM, Boyle KA, Madden JF, Dickinson SA, Jobling P, Callister RJ, Hughes DI, Graham BA (2015) Functional heterogeneity of calretinin-expressing neurons in the mouse superficial dorsal horn: implications for spinal pain processing. J Physiol 593:4319–4339. 10.1113/JP270855 26136181 PMC4594251

[B62] Stensrud MJ, Chaudhry FA, Leergaard TB, Bjaalie JG, Gundersen V (2013) Vesicular glutamate transporter-3 in the rodent brain: vesicular colocalization with vesicular γ-aminobutyric acid transporter. J Comp Neurol 521:3042–3056. 10.1002/cne.23331 23633129

[B63] Todd AJ (1996) GABA and glycine in synaptic glomeruli of the rat spinal dorsal horn. Eur J Neurosci 8:2492–2498. 10.1111/j.1460-9568.1996.tb01543.x 8996798

[B64] Todd AJ (2010) Neuronal circuitry for pain processing in the dorsal horn. Nat Rev Neurosci 11:823–836. 10.1038/nrn2947 21068766 PMC3277941

[B65] Todd AJ (2017) Identifying functional populations among the interneurons in laminae I-III of the spinal dorsal horn. Mol Pain 13:1744806917693003. 10.1177/1744806917693003 28326935 PMC5315367

[B66] Todd AJ, McGill MM, Shehab SAS (2000) Neurokinin 1 receptor expression by neurons in laminae I, III and IV of the rat spinal dorsal horn that project to the brainstem. Eur J Neurosci 12:689–700. 10.1046/j.1460-9568.2000.00950.x 10712649

[B67] Todd AJ, Hughes DI, Polgár E, Nagy GG, Mackie M, Ottersen OP, Maxwell DJ (2003) The expression of vesicular glutamate transporters VGLUT1 and VGLUT2 in neurochemically defined axonal populations in the rat spinal cord with emphasis on the dorsal horn. Eur J Neurosci 17:13–27. 10.1046/j.1460-9568.2003.02406.x 12534965

[B68] Usoskin D, Furlan A, Islam S, Abdo H, Lönnerberg P, Lou D, Hjerling-Leffler J, Haeggström J, Kharchenko O, Kharchenko PV, Linnarsson S, Ernfors P (2015) Unbiased classification of sensory neuron types by large-scale single-cell RNA sequencing. Nat Neurosci 18:145–153. 10.1038/nn.3881 25420068

[B69] Vallbo Å, Olausson H, Wessberg J, Norrsell U (1993) A system of unmyelinated afferents for innocuous mechanoreception in the human skin. Brain Res 628:301–304. 10.1016/0006-8993(93)90968-s 8313159

[B70] Weston MC, Nehring RB, Wojcik SM, Rosenmund C (2011) Interplay between VGLUT isoforms and endophilin A1 regulates neurotransmitter release and short-term plasticity. Neuron 69:1147–1159. 10.1016/j.neuron.2011.02.002 21435559

[B71] Willis W, Coggeshall R (2004) Sensory mechanisms of the spinal cord. New York: Springer.

[B72] Woodbury CJ, Ritter AM, Koerber HR (2000) On the problem of lamination in the superficial dorsal horn of mammals: a reappraisal of the substantia gelatinosa in postnatal life. J Comp Neurol 417:88–102. 10.1002/(SICI)1096-9861(20000131)417:1<88::AID-CNE7>3.0.CO;2-U 10660890

[B73] Yasaka T, Tiong SY, Polgár E, Watanabe M, Kumamoto E, Riddell JS, Todd AJ (2014) A putative relay circuit providing low-threshold mechanoreceptive input to lamina I projection neurons via vertical cells in lamina II of the rat dorsal horn. Mol Pain 10:3. 10.1186/1744-8069-10-3 24433581 PMC3897975

[B74] Zotterman Y (1939) Touch, pain and tickling: an electro-physiological investigation on cutaneous sensory nerves. J Physiol 95:1–28. 10.1113/jphysiol.1939.sp003707 16995068 PMC1393960

